# Long non-coding RNA MALAT1 facilitates the tumorigenesis, invasion and glycolysis of multiple myeloma via miR-1271-5p/SOX13 axis

**DOI:** 10.1007/s00432-020-03127-8

**Published:** 2020-01-18

**Authors:** Na Liu, Song Feng, Huanhuan Li, Xiaoguang Chen, Songting Bai, Yufeng Liu

**Affiliations:** Department of Pediatrics, The First Affiliated Hospital of Zhengzhou University, No. 1 Jianshe East Road, Erqi District, Zhengzhou, 450052 Henan China

**Keywords:** MALAT1, Multiple myeloma, Glycolysis, miR-1271-5p, SOX13

## Abstract

**Background:**

Long non-coding RNAs (lncRNAs) play crucial roles in the regulation and treatment of multiple myeloma (MM). The objective of this research was to study the functional mechanism of metastasis-associated lung adenocarcinoma transcript 1 (MALAT1) in MM.

**Methods:**

MALAT1, microRNA-1271-5p (miR-1271-5p), and SRY-Box 13 (SOX13) levels were examined by quantitative real-time polymerase chain reaction (qRT-PCR). Cell viability, apoptosis, and invasion were respectively assayed using 3-(4, 5-dimethylthiazol-2-y1)-2, 5-diphenyl tetrazolium bromide (MTT), flow cytometry, and transwell assay. Glycolysis was evaluated by glucose consumption, lactate production, ATP/ADP ratio, and the detection of related enzymes. Associated proteins were measured using Western blot. Target relation was verified via dual-luciferase reporter assay. Xenograft tumor assay was implemented to study the influence of MALAT1 on MM in vivo.

**Results:**

The up-regulation of MALAT1 and the down-regulation of miR-1271-5p were found in MM serums and cells. MALAT1 knockdown suppressed cell viability, invasion, and glycolysis while expedited cell apoptosis in MM cells. MALAT1 directly targeted miR-1271-5p and miR-1271-5p depression reverted the effects of MALAT1 knockdown on MM cells. SOX13 was a target of miR-1271-5p and SOX13 overexpression weakened the effects of miR-1271-5p on MM. MALAT1 indirectly modulated SOX13 expression through targeting miR-1271-5p. MALAT1 down-regulation inhibited MM growth by miR-1271-5p/SOX13 axis in vivo.

**Conclusion:**

LncRNA MALAT1 expedited MM tumorigenesis, invasion, and glycolysis via miR-1271-5p/SOX13 axis. MALAT1 might contribute to the therapy of MM as a promising indicator.

## Introduction

Multiple myeloma (MM) is defined as a B-cell malignancy with the characteristic of abnormal expansion and abundance of plasma cells in the bone marrow to trigger extramedullary disease (Dimopoulos and Terpos 2010). As the second most frequent hematological malignancy, MM has a comparatively higher incidence and mortality (Laubach et al. 2011). Although significant advancements have been developed with conventional chemotherapy, stem cell transplantation, and nanomedicine (Detappe et al. 2018; Laubach et al. 2011), the 5-year survival rate remains gloomy for MM patients. The urgent affair is to seek novel molecules to confront the aggressive MM.

Long non-coding RNAs (lncRNAs) belong to ncRNAs characterized by the length of more than 200 nucleotides and the absence of protein-coding ability (Ponting et al. 2009). LncRNAs can regulate cellular phenotypical changes in various types of cancers (Li and Chen 2016). Recently, Pan et al. reported the high expression of H19 in MM patients and cells, speculating the potential of H19 as a diagnostic biomarker for MM (Pan et al. 2018). Zhang et al. declared that UCA1 enhanced cell growth of MM cells (Zhang et al. 2018). Also, metastasis-associated lung adenocarcinoma transcript 1 (MALAT1) was up-regulated in MM previously (Gu et al. 2017). But the precise role and mechanism of MALAT1 in MM have not been fully illuminated.

MicroRNAs (miRNAs) are a genre of ncRNAs marked by the interacting with the 3′ untranslated region (3′UTR) of the messenger RNA (mRNA) to regulate gene expression (Matoulkova et al. 2012). miRNAs usually play anti-tumor roles in MM according to previous studies. Liu et al. reported that miR-215-5p acted as an anticancer factor in MM via targeting RUNX1 and blocking the PI3K/AKT/mTOR pathway (Liu et al. 2019b) and miR-186 was shown to suppress cell proliferation in MM (Deng et al. 2019). A recent study revealed the low expression of miR-1271-5p in MM (Yang and Chen 2019). SRY-Box 13 (SOX13), a member of the Sry-related high-mobility group box (Sox) transcription factor family, was testified to participate in the progression of several tumors, such as glioma (He et al. 2019) and gastric carcinoma (Bie et al. 2019). Xu et al. stated the overexpression of SOX13 in MM (Xu et al. 2018). Nevertheless, the relation among MALAT1, miR-1271-5p, and SOX13 in MM remains obscure.

In this report, we explored the functional role of MALAT1 in cell viability, apoptosis, invasion, and glycolysis of MM cells. The regulatory mechanism among MALAT1, miR-1271-5p, and SOX13 in MM was investigated.

## Materials and methods

### Patients and serum collection

Serum samples were collected from MM patients (*n* = 30) and healthy donors (*n* = 30) in the First Affiliated Hospital of Zhengzhou University. After centrifugation at 3000×*g *for 15 min, the supernatant serum was transferred into an RNase-free Eppendorf tube and immediately conserved in − 80 °C ultra-low temperature refrigerator for use. All patients and healthy subjects were completely informed and signed the written informed consents. This research got authorization from the Ethic Committee of the First Affiliated Hospital of Zhengzhou University.

### Cell culture

Human normal plasma cell line (nPCs) was acquired from Fenghui Biotechnology Co., Ltd (Changsha, China) and MM cell lines (NCI-H929 and OPM-2) were bought from Xiangf Biosciences Co., Ltd. (Shanghai, China). All cells were cultivated in Roswell Park Memorial Institute-1640 (RPMI-1640; Corning Life Sciences, Corning, NY, USA) added with 10% fetal bovine serum (FBS; Serapro, Naila, Germany), and 1% penicillin–streptomycin mixed solution (Transgen, Beijing, China) in humidified air with 5% CO_2_ at 37 °C.

### Quantitative real-time polymerase chain reaction (qRT-PCR)

The qRT-PCR assay was administrated applying SYBR Green PCR Kit (Applied Biosystems, Foster City, CA, USA) through the ABI Prism 7500 sequence detection system (Applied Biosystems) as previously reported (Jin et al. 2019). The relative expression levels were analyzed via the 2^−∆∆Ct^ approach (Livak and Schmittgen 2001) using glyceraldehyde-3-phosphate dehydrogenase (GAPDH) for normalizing MALAT1 or SOX13 and U6 as the internal reference of miR-1271-5p. Primers sequences used in this report were as follows: MALAT1 (Forward: 5′-AAAGCAAGGTCTCCCCACAA-3′, Reverse: 5′-GGTCTGTGCTAGATCAAAAGGCA-3′); miR-1271-5p (Forward: 5′-CAGCACTTGGCACCTAGCA-3′, Reverse: 5′-TATGGTTGTTCTCCTCTCTGTCTC-3′); SOX13 (Forward: 5′-CTGGACTTCAACCGAAATTTGA-3′, Reverse: 5′-GTTCCTTCCTAGAAACCTCTCC-3′); GAPDH (Forward: 5′-GCATCCTGGGCTACACTG-3′, Reverse: 5′-TGGTCGTTGAGGGCAAT-3′); U6 (Forward: 5′-GCTTCGGCAGCACATATACTAAAAT-3′, Reverse: 5′-CGCTTCACGAATTTGCGTGTCAT-3′).

### Transient transfection

Cell transient transfection was conducted using Lipofectamine3000 reagent (Invitrogen, Carlsbad, CA, USA) complying with the manufacturer’s instruction. Small interfering RNA (siRNA) against MALAT1 (si-MALAT1), miR-1271-5p mimic and inhibitor (miR-1271-5p and anti-miR-1271-5p) and respective negative controls (si-NC, miR-NC and anti-miR-NC) were purchased from Ribobio (Guangzhou, China). The sequence of SOX13 was cloned into pcDNA vector (Invitrogen) to construct the overexpression vector pcDNA-SOX13 (SOX13) with pcDNA as the negative control.

### 3-(4, 5-dimethylthiazol-2-y1)-2, 5-diphenyl tetrazolium bromide (MTT) assay

Firstly, a total number of 2 × 10^3^ MM cells were plated into 96-well plates overnight. Following transfection for 24 h, 48 h and 72 h, 20 µL MTT (Sangon Biotech, Shanghai, China) were pipetted into the wells, mixing with cells for another 4 h. Next, the supernatant was removed and cells were incubated with dimethyl sulfoxide (DMSO; Sangon Biotech) with 200 µL per well. Ten minutes later, the optical density (OD) value at 490 nm was determined by a microplate reader, which represented the viability of MM cells. The un-transfected cells were used as the transfection control group.

### Flow cytometry

Transfected MM cells were washed by pre-cooled phosphate buffer solution (PBS; Corning Life Sciences) and centrifugally harvested at 2000 rpm for 10 min. Cell pellets were resuspended in 500 μL Binding buffer, then mixed with respective 5 μL Annexin V-fluorescein isothiocyanate (Annexin V-FITC), and propidium iodide (PI) (BD Biosciences, San Diego, CA, USA) for 20 min in the dark. Eventually, apoptotic cells could be distinguished through a flow cytometer (BD Biosciences) and apoptosis rate was calculated.

### Transwell invasion assay

At the beginning, the low side of the upper chamber of the transwell chamber (Corning Life Sciences) was firstly enveloped with matrigel (Corning Life Sciences). Then cell suspension in serum-free medium was pipetted into the upper chamber, accompanying with the adding of RIPM-1640 containing 10% FBS into the lower chamber. Subsequently, cells were fixated using methanol (Sangon Biotech) and colored with crystal violet (Sangon Biotech) 48 h later. Ultimately, invaded cells were counted through a microscope after uninvaded cells were wiped off with a wet cotton swab.

### Detection of glucose consumption, lactate production, and ATP/ADP ratio

Cell supernatant was collected post-transfection for 48 h, then glucose and lactate levels were severally examined via Glucose Uptake Colorimetric Assay Kit and Lactate Colorimetric Assay Kit (Biovision, San Francisco, CA, USA) in line with the producers’ directions. ATP/ADP ratio was measured by ApoSENSOR™ ADP/ATP Ratio Bioluminescence Assay Kit (Biovision). Briefly, 1 × 10^4^ cells were inoculated into the luminometer plate and Nucleotide Releasing Buffer was added. Then the values of wells were read at 1 min (A) and 10 min (B) after adding with 1 μL ATP Monitoring Enzyme. Sample values were recorded (C) following the addition of ADP Converting Enzyme. ATP/ADP ratio was calculated according to the formula: A/(C − B).

### Western blot

After the extraction of proteins from serums and cells via radioimmunoprecipitation assay (RIPA) lysis solution (Beyotime, Shanghai, China), 60 μg quantified proteins were isolated on 10% sodium dodecyl sulfate–polyacrylamide gel for 2 h, followed by the transferring of proteins onto the polyvinylidene fluoride membranes (Beyotime). Then, 5% skim milk (Beyotime) was applied for blocking membranes for 3 h, which were incubated with primary antibodies from Abcam (Cambridge, United Kingdom): anti-Hexokinase 2 (anti-HK2; ab209847, 1:1000), anti-Glucose Transporter 1 (anti-GLUT1; ab115730, 1:1000), anti-SOX13 (ab198921, 1:1000) and anti-GAPDH (ab9485, 1:3000) overnight at 4 °C Following the incubation of secondary antibody (Abcam, ab205718, 1:5000) for 1 h, the immunoreactive signals were assayed through enhanced chemiluminescence reagent (Abcam), and the gray levels were analyzed via the Image J software (NIH, Bethesda, MD, USA).

### Dual-luciferase reporter assay

The bioinformatics analysis was executed by Starbase v2.0. Dual-luciferase reporter assay was used for validating the combination between miR-1271-5p and MALAT1 or SOX13. The sequences of wild-type (WT) MALAT1 and 3′UTR of SOX13 (with the binding sites for miR-1271-5p) were inserted into pmirGLO vector (Promega, Madison, WI, USA) to form WT luciferase reporters WT-MALAT1 and SOX13 3′UTR-WT. After the complementary sites for miR-1271-5p in MALAT1 and SOX13 3′UTR were mutated, the mutant-type (MUT) reporters MUT-MALAT1 and SOX13 3′UTR-MUT were constructed. Afterwards, MM cells were respectively co-transfected with above reporters and miR-1271-5p or miR-NC. Finally, the dual-luciferase reporter assay system (Promega) was employed for the detection of luciferase activities from cell lysates. Firefly luciferase activity was standardized by renilla activity and the ratio of firefly/renilla was considered as the relative luciferase activity.

### Xenograft tumor assay

Short hairpin RNA (shRNA) against MALAT1 (sh-MALAT1) was synthesized by GenePharma (Shanghai, China) to establish stably transfected cells via lentivirus mediation with sh-NC as the negative control. After the purchase of BALB/c nude mice (6-week-old) from Vital River Laboratory Animal Technology (Beijing, China), xenograft tumor model was established through the subcutaneous injection into mice with NCI-H929 cells (5 × 10^6^ cells/mice) stably expressed sh-MALAT1 or sh-NC (seven mice per group). Tumor volume (length × width^2^ × 0.5) was recorded every 4 days post-injection 8 days. After 28 days, tumor tissues were excised from euthanized mice and weighed. And the levels of MALAT1, miR-1271-5p, and SOX13 in tissues were measured. This animal experiment was ratified by the Animal Ethics Committee of the First Affiliated Hospital of Zhengzhou University.

### Statistical analysis

Assays in this study were implemented by three repetitive parallels and data were expressed as mean ± standard deviation (SD). SPSS 19.0 was used for data analysis and statistical processing. Graphing was performed in GraphPad Prism 7. The linear relationship was analyzed via Spearman’s correlation coefficient. The difference comparison was analyzed by Student’s *t* test or one-way analysis of variance (ANOVA) followed by Tukey’s test. Difference was defined as statistically significant with* P* < 0.05.

## Results

### MALAT1 was up-regulated while miR-1271-5p was down-regulated in MM serums and cells

**Fig. 1:**
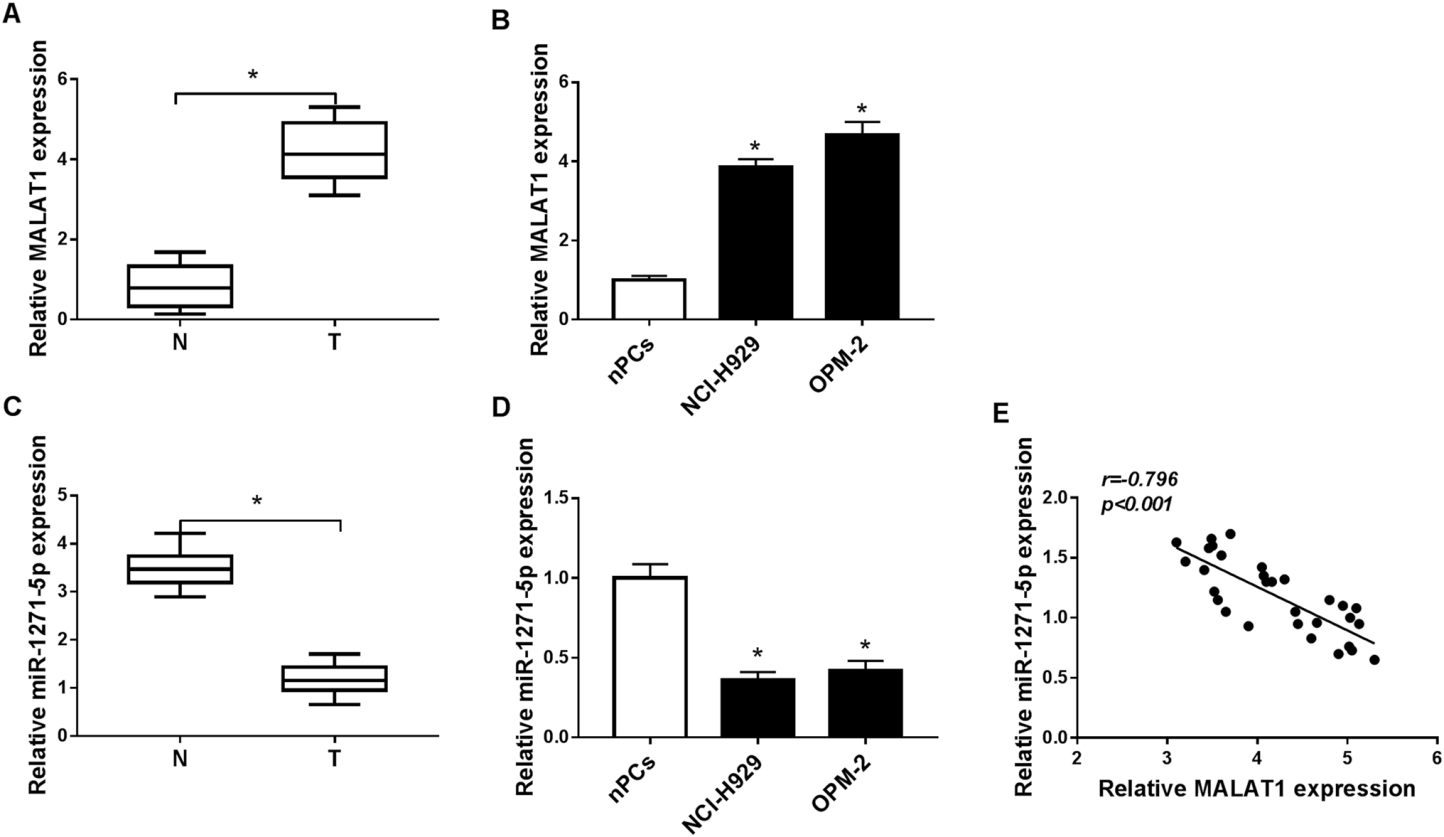
MALAT1 was up-regulated while miR-1271-5p was down-regulated in MM serums and cells. (**a**–**d**) The expression levels of MALAT1 (**a**, **b**) and miR-1271-5p (**c**, **d**) were determined in MM serums and cells by qRT-PCR. **e** The relation between MALAT1 and miR-1271-5p in MM serum specimens via Spearman’s correlation coefficient. *P < 0.05

We first assayed the expression of MALAT1 in MM by qRT-PCR. Compared with normal serum samples, MALAT1 level was significantly increased in MM serums (Fig. 1a). Also, MALAT1 was expressed higher in NCI-H929 and OPM-2 cells than that in normal nPCs cells (Fig. 1b). Then we found the inverse down-regulation of miR-1271-5p in MM serums (Fig. 1c) and cells (Fig. 1d) by comparison with normal serums and cells. After the analysis of Spearman’s correlation coefficient, a negative relation (*r* =  − 0.796, *P* < 0.001) between MALAT1 and miR-1271-5p was exhibited in MM serums (Fig. 1e). These results indicated MALAT1 was enriched while miR-1271-5p was decreased in MM.

### Knockdown of MALAT1 refrained cell viability, invasion and glycolysis but induced cell apoptosis in MM cells

**Fig. 2:**
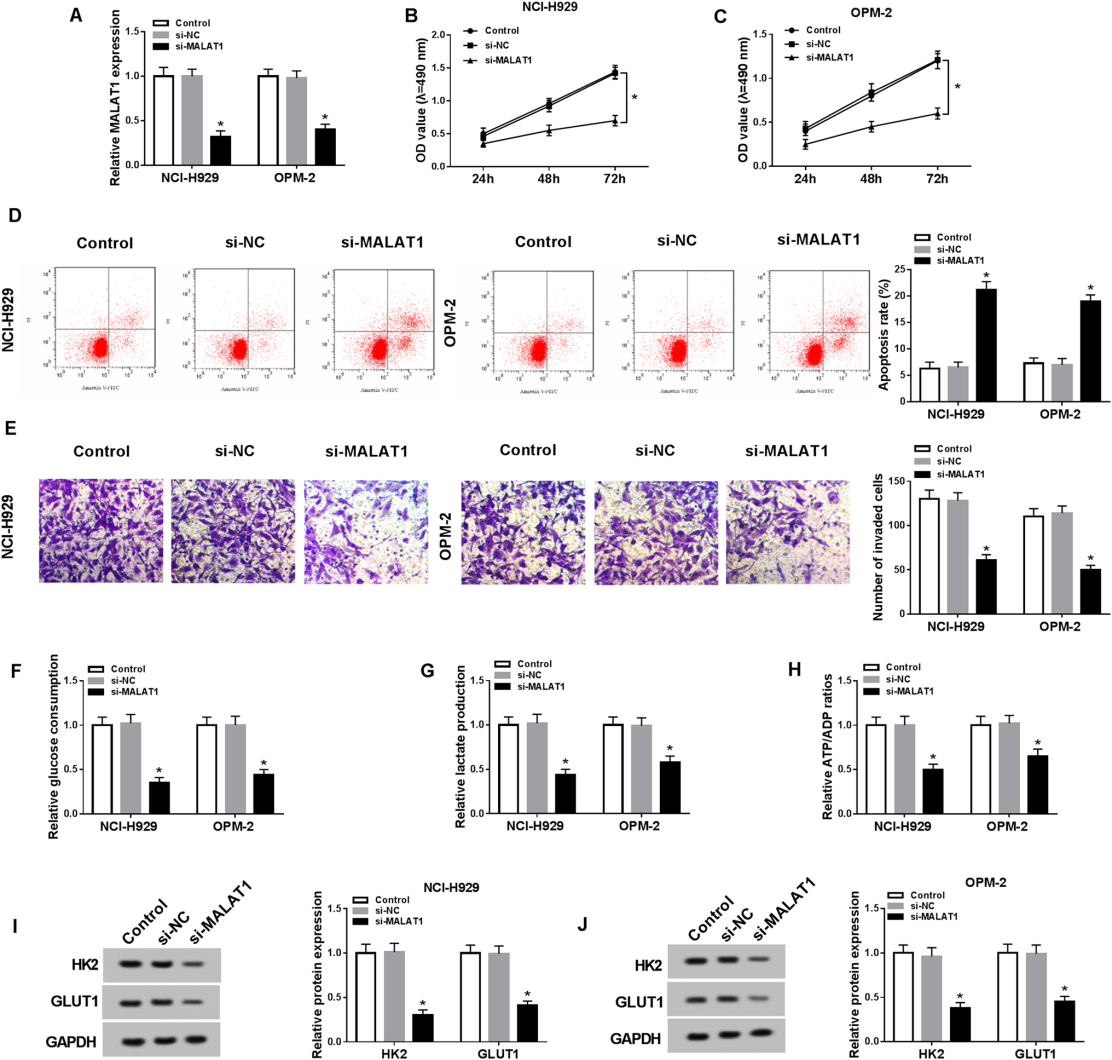
Knockdown of MALAT1 refrained cell viability, invasion, and glycolysis but induced cell apoptosis in MM cells. **a** The knockdown efficiency of si-MALAT1 was assayed using qRT-PCR. **b**, **c** Cell viability was determined via MTT in NCI-H929 and OPM-2 cells transfected with si-MALAT1 or si-NC and un-transfected cells. **d**, **e** Flow cytometry and transwell assay were severally applied for the detection of cell apoptosis (**d**) and invasion (**e**). **f**–**j** Glycolysis was evaluated through the glucose consumption (**f**), lactate production (**g**), ATP/ADP ratio (**h**) and the examination of glycolysis-associated enzymes by Western blot (I and J). *P < 0.05

To investigate the potential function of MALAT1 in MM, si-MALAT1 was synthesized to disturb the expression of MALAT1 and the interference was successful in NCI-H929 and OPM-2 cells compared to si-NC and control groups (Fig. 2a). Then we applied further experiments to assess cellular processes of MMC cells. MTT showed that the OD value of si-MALAT1 group was obviously lower than that of si-NC group in NCI-H929 (Fig. 2b) and OPM-2 (Fig. 2c) cells. si-MALAT1 transfection caused the promotion of apoptosis rate (Fig. 2d) but the lessening of invaded cells number (Fig. 2e). Moreover, glycolysis process was evaluated. The glucose consumption (Fig. 2f) and lactate production (Fig. 2g) levels were fewer in NCI-H929 and OPM-2 cells transfected with si-MALAT1 compared to the si-NC group. The ATP/ADP ratio also declined after knockdown of MALAT1 (Fig. 2h). Meanwhile, the glycolysis-associated enzymes were examined by Western blot, in which the protein levels of HK2 and GLUT1 were notably repressed following si-MALAT1 transfection (Fig. 2i, j), verifying the inhibition of cellular glycolysis by MALAT1 knockdown again. In short, MALAT1 down-regulation repressed cell viability, invasion, and glycolysis but promoted apoptosis in MM cells.

### MALAT1 targeted miR-1271-5p and miR-1271-5p inhibition returned the effects of MALAT1 knockdown on MM cells

**Fig. 3:**
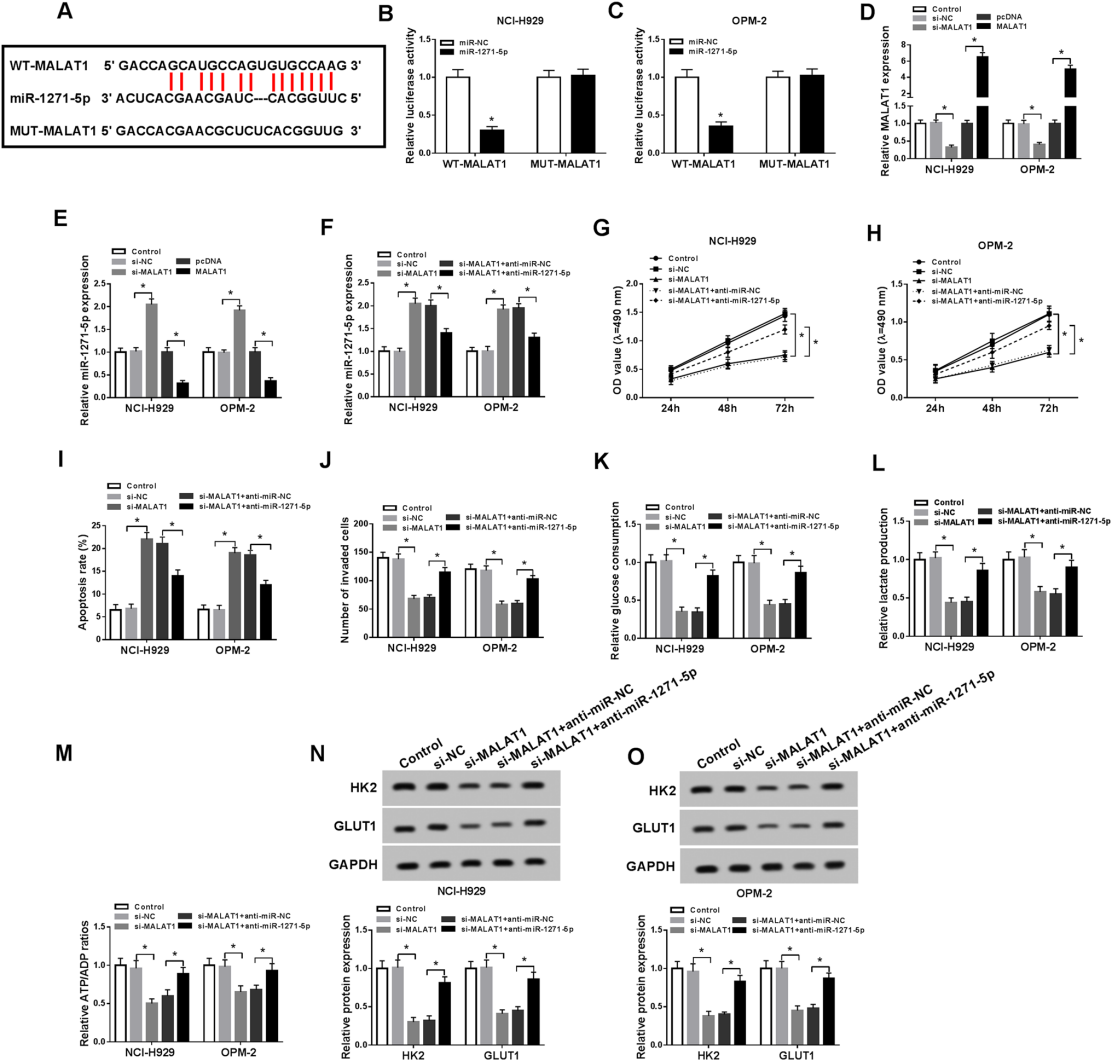
MALAT1 targeted miR-1271-5p and miR-1271-5p inhibition returned the effects of MALAT1 knockdown on MM cells. **a** Starbase v2.0 was performed for predicting the miRNA target of MALAT1. **b**, **c** The interaction between MALAT1 and miR-1271-5p was confirmed using the dual-luciferase reporter assay. **d**, **e** QRT-PCR was used to determine the expression of MALAT1 (**d**) and miR-1271-5p (**e**) after transfection with si-MALAT1, MALAT1 or relative controls. **f** The miR-1271-5p level was measured by qRT-PCR in NCI-H929 and OPM-2 cells transfected with si-MALAT1, si-MALAT1 + anti-miR-1271-5p or corresponding controls. **g**, **h** MTT was implemented to examine cell viability. **i**, **j** Cell apoptosis (**i**) and invasion (**j**) were assayed via flow cytometry and transwell assay, separately. **k**–**o** The glucose consumption (**f**), lactate production (**g**), ATP/ADP ratio (**h**) and the examination of glycolysis-associated enzymes by Western blot (**i**, **j**) were used to assess the glycolysis process. *P < 0.05

LncRNAs combine with miRNAs generally (Peng et al. 2018; Su et al. 2019; Wang et al. 2017). After the bioinformatics analysis by Starbase v2.0, we found MALAT1 contained the combinative sites for miR-1271-5p (Fig. 3a), speculating that miR-1271-5p might be a target of MALAT1. Then, miR-1271-5p transfection strikingly decreased the luciferase activity of WT-MALAT1 group but not in MUT-MALAT1 group in NCI-H929 and OPM-2 cells (Fig. 3b, c), affirming the combination between MALAT1 and miR-1271-5p. After MALAT1 was successfully knocked down and overexpressed (Fig. 3d), miR-1271-5p expression was assayed. The results manifested MALAT1 knockdown heightened the miR-1271-5p expression but MALAT1 overexpression displayed the contrary phenomenon (Fig. 3e). To explore the regulatory relation between MALAT1 and miR-1271-5p in MM, NCI-H929 and OPM-2 cells were transfected with si-MALAT1, si-MALAT1 + anti-miR-1271-5p or matched controls. As shown in Fig. 3f, miR-1271-5p inhibition rescued the rising of miR-1271-5p induced by MALAT1 knockdown. MTT proved that si-MALAT1-induced inhibition of cell viability was ameliorated after miR-1271-5p was down-regulated (Fig. 3g, h) a The stimulative effect on cell apoptosis (Fig. 3i) and inhibitory effect on cell invasion (Fig. 3j) caused by si-MALAT1 were alleviated by miR-1271-5p inhibitor. Also, the down-regulation of miR-1271-5p reverted the suppression of glucose consumption (Fig. 3k), lactate production, (Fig. 3l) and ATP/ADP ratio (Fig. 3m), as well as the restraint of HK2 and GLUT1 protein levels (Fig. 3n, o) in NCI-H929 and OPM-2 cells transfected with si-MALAT1. All in all, MALAT1 targeted miR-1271-5p and miR-1271-5p down-regulation relieved the effects of MALAT1 knockdown on MM cells.

### MiR-1271-5p directly targeted SOX13

**Fig. 4:**
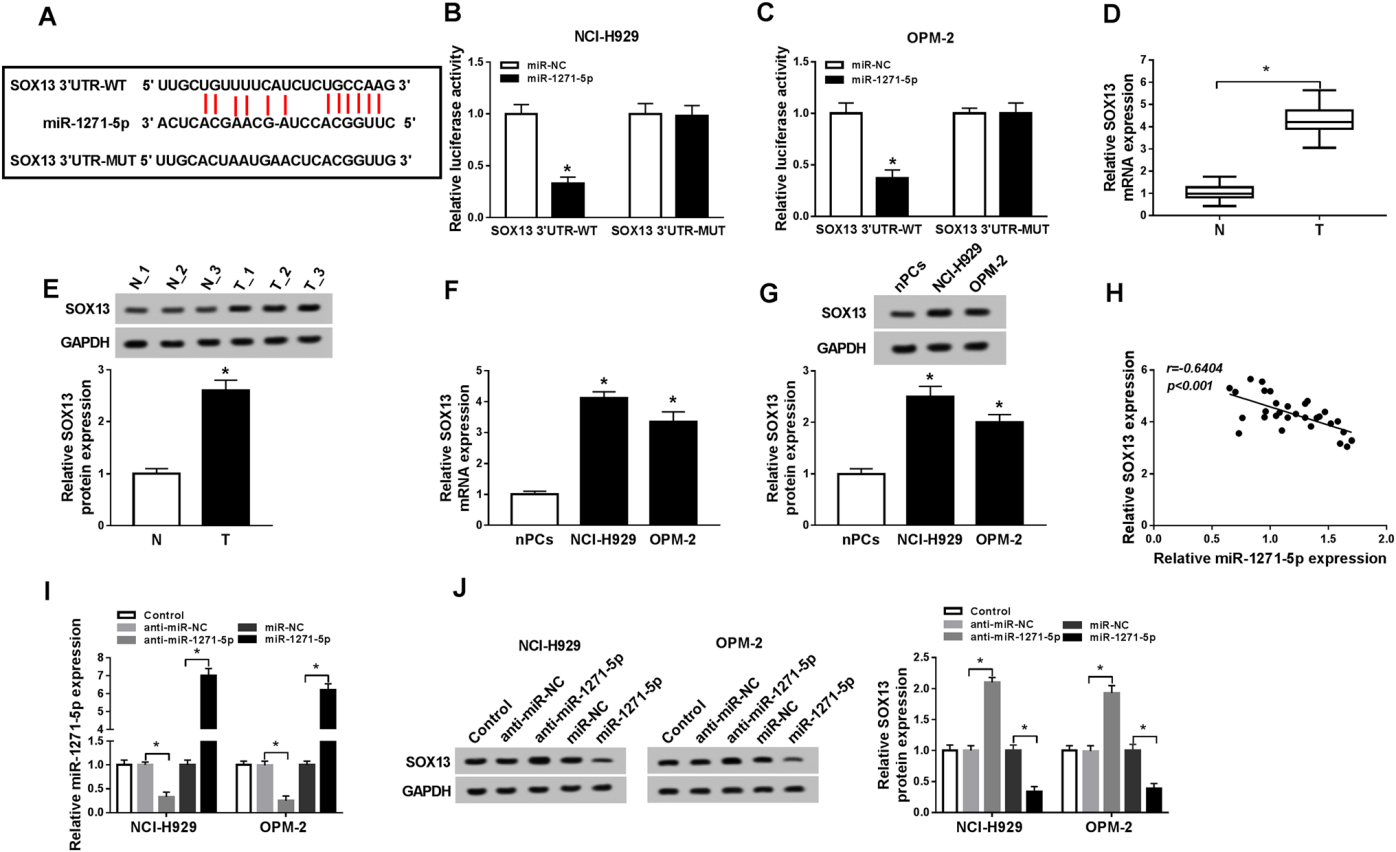
MiR-1271-5p directly targeted SOX13. **a** The target gene of miR-1271-5p was predicted by Starbase v2.0. **b**, **c** Dual-luciferase reporter assay was administered to validate the combination between miR-1271-5p and SOX13. **d**–**g** The SOX13 level in MM serums (**d**, **e**) and cells (**f**, **g**) was assayed using qRT-PCR and Western blot. **h** Spearman’s correlation coefficient was carried out to analyze the relation between miR-1271-5p and SOX13 in MM serum samples. **i** The inhibitory effect of anti-miR-1271-5p and overexpressed effect of miR-1271-5p on miR-1271 level were assessed through qRT-PCR. **j** The protein level of SOX13 was measured by Western blot in MM cells transfected with anti-miR-1271-5p, miR-1271-5p and matched controls. *P < 0.05

miRNAs usually interact with the 3′UTR of downstream gene (Li et al. 2018; Wei et al. 2017, 2019). Starbase v2.0 revealed that SOX13 had the mutual binding sites for miR-1271-5p (Fig. 4a), implying that SOX13 might be a target candidate of miR-1271-5p. Dual-luciferase reporter assay further validated that the luciferase activity in SOX13 3′UTR-WT group was descended by miR-1271-5p, but there was no evident change in SOX13 3′UTR-MUT group (Fig. 4b, c). Subsequently, we found the up-regulation of SOX13 mRNA and protein levels in MM serums (Fig. 4d, e) and cells (Fig. 4f, g) in comparison to normal serums and cells aThere was a negative association (*r* = − 0.6404, *P* < 0.001) between miR-1271-5p and SOX13 in MM serum samples (Fig. 4h). QRT-PCR analysis showed the inhibitory effect of anti-miR-1271-5p and overexpressed effect of miR-1271-5p on miR-1271-5p expression were great (Fig. 4i). miR-1271-5p inhibitor elevated SOX13 protein level but miR-1271-5p overexpression triggered the down-regulation of SOX13 expression (Fig. 4j). Therefore, miR-1271-5p negatively regulated SOX13 level.

### Overexpression of SOX13 relieved the miR-1271-5p-induced effects on MM cells

**Fig. 5:**
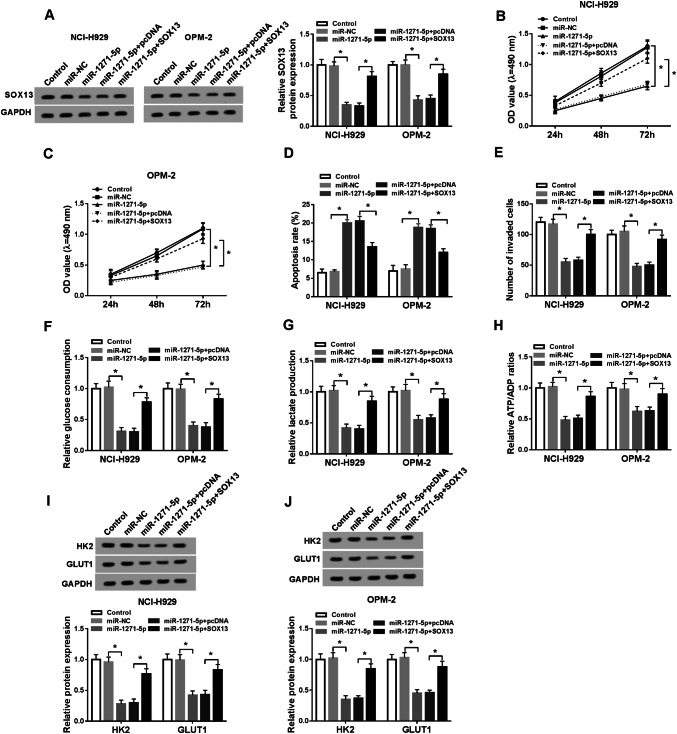
Overexpression of SOX13 relieved the miR-1271-5p-induced effects on MM cells. **a** Western blot was used to determine the SOX13 protein expression in NCI-H929 and OPM-2 cells transfected with miR-1271-5p, miR-1271-5p + SOX13 or corresponding controls. **b**, **c** The evaluation of cell viability was executed via MTT assay. **d**, **e** Apoptosis rate (**d**) and invaded cells (**e**) were individually measured using flow cytometry and transwell assay. **f**–**j** The assessment of glycolysis was implemented by glucose consumption **f**, lactate production (**g**), ATP/ADP ratio (**h**) and the detection of glycolysis-associated enzymes by Western blot (**i**, **j**). *P < 0.05

The regulatory relation between miR-1271-5p and SOX13 was investigated after transfection with miR-1271-5p, miR-1271-5p + SOX13 or respective controls. Firstly, we observed that the inhibition of SOX13 protein expression induced by miR-1271-5p was abated through SOX13 up-regulation (Fig. 5a). Then MTT assay presented that the intervention of miR-1271-5p evoked the refrainment of cell viability, which was abolished by the overexpression of SOX13 (Fig. 5b, c). Additionally, transfection of SOX13 abrogated the promotion of cell apoptosis (Fig. 5d) and the repression of invasion (Fig. 5e) caused by miR-1271-5p. Also, miR-1271-5p led to the reduction of glucose consumption (Fig. 5f), lactate production (Fig. 5g), ATP/ADP ratio, (Fig. 5h) and the protein levels of HK2 and GLUT1 (Fig. 5i, j), whereas ectopic overexpression of SOX13 reverted these effects. Above results demonstrated that SOX13 overexpression reversed the miR-1271-5p-induced inhibition of cell viability, invasion and glycolysis but the enhancement of apoptosis in MM cells.

### MALAT1 modulated SOX13 expression via targeting miR-1271-5p

**Fig. 6:**
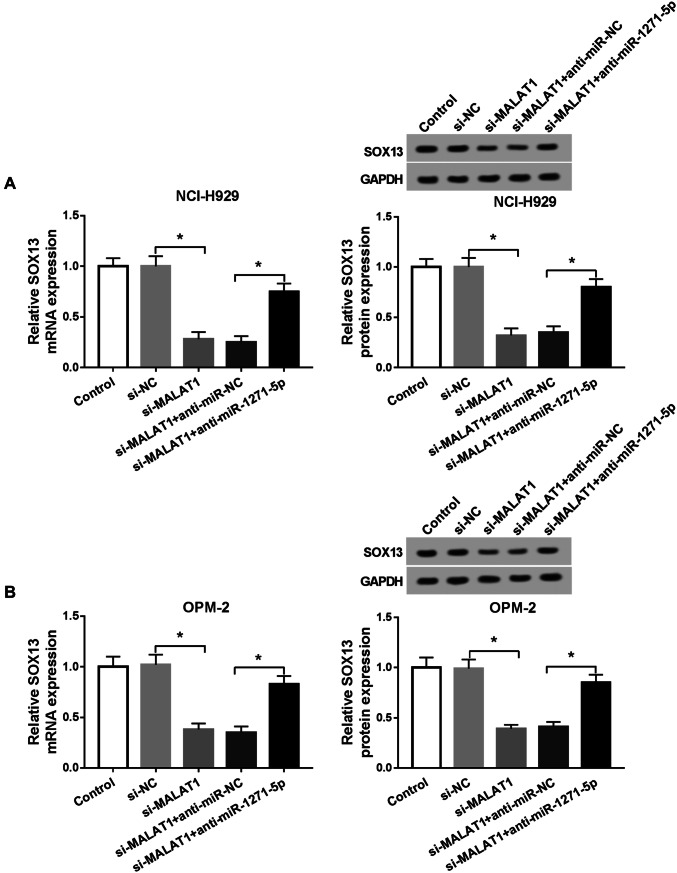
MALAT1 modulated SOX13 expression via targeting miR-1271-5p. **a**, **b** The mRNA and protein levels of SOX13 were examined via qRT-PCR and Western blot in NCI-H929 (**a**) and OPM-2 (**b**) cells transfected with si-MALAT1, si-MALAT1 + anti-miR-1271-5p or matched controls. *P < 0.05

We transfected si-MALAT1, si-MALAT1 + anti-miR-1271-5p or relative controls into NCI-H929 and OPM-2 cells for the purpose of studying the relation between MALAT1 and SOX13. As Fig. 6a, b described, transfection of si-MALAT1 resulted in the restraint of SOX13 mRNA and protein levels, which was counteracted following the inhibition of miR-1271-5p expression in NCI-H929 and OPM-2 cells. Therefore, MALAT1 could regulate the level of SOX13 through the indirect interaction with miR-1271-5p.

### Down-regulation of MALAT1 reduced MM growth via miR-1271-5p/SOX13 axis in vivo

**Fig. 7:**
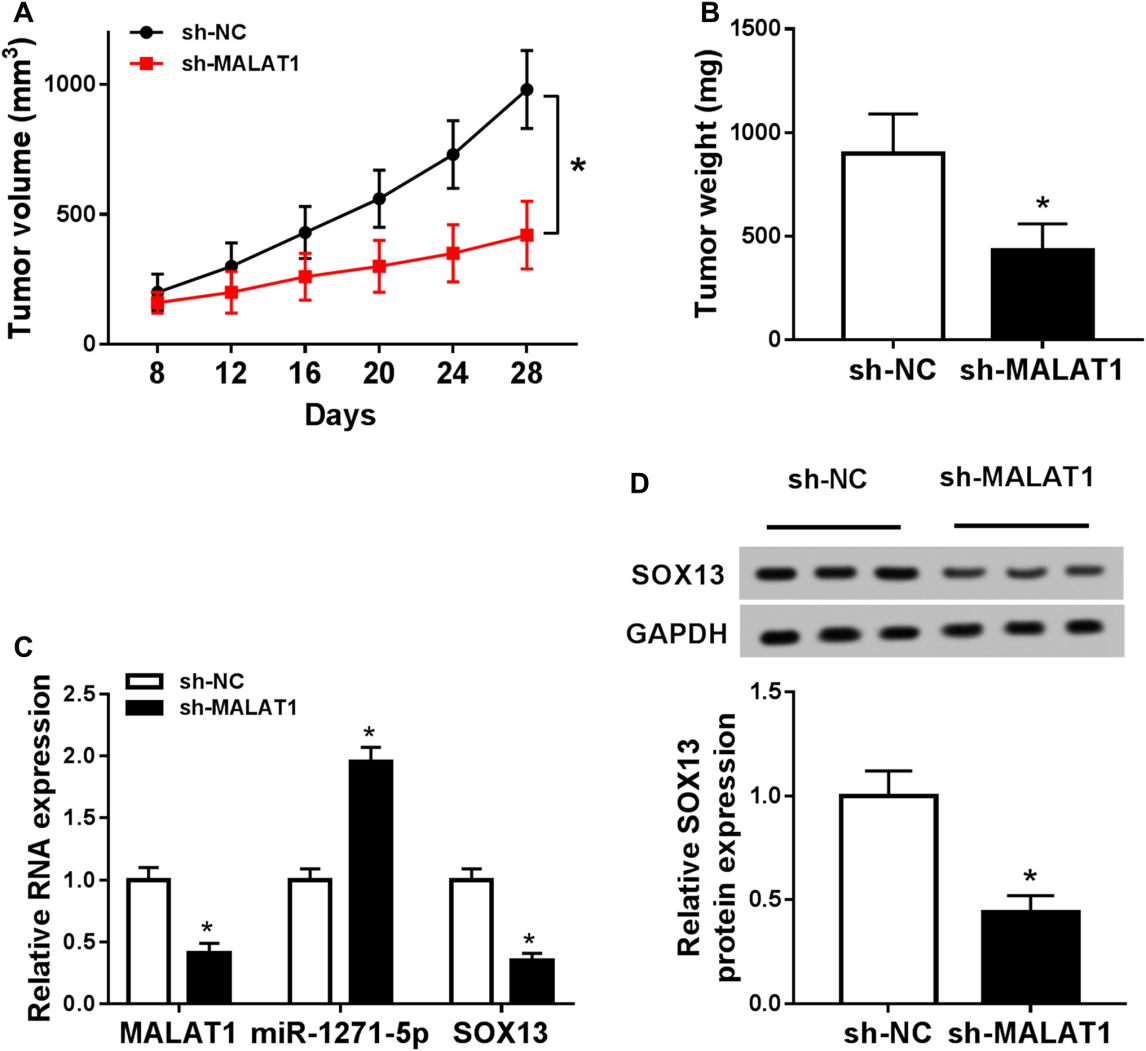
Down-regulation of MALAT1 reduced MM growth via miR-1271-5p/SOX13 axis in vivo. **a**, **b** Tumor volume (**a**) and weight (**b**) were recorded and measured in sh-MALAT1 and sh-NC groups. **c** The levels of MALAT1, miR-1271-5p, and SOX13 mRNA were assayed by qRT-PCR in excised tissues. **d** Western blot was applied for examining the SOX13 protein level in vivo. *P < 0.05

The xenograft tumor model was established to ascertain the impact of MALAT1 on MM in vivo. Through the measurement and record, we found that tumor volume (Fig. 7a) and weight (Fig. 7b) declined in sh-MALAT1 group contrasted to sh-NC group. Moreover, knockdown of MALAT1 caused the down-regulation of MALAT1 and SOX13, as well as the increase of miR-1271-5p expression in excised tissues (Fig. 7c). After the analysis of Western blot, the SOX13 protein level was lower in sh-MALAT1 group than that in sh-NC group (Fig. 7d). These data manifested that MALAT1 knockdown inhibited MM growth by miR-1271-5p/SOX13 axis in vivo.

## Discussion

The advanced therapies of MM have been developed in medical research, but the molecular pathogenesis mechanism underlying the tumorigenesis of MM has not completely addressed up to now. Herein, lncRNA MALAT1 was up-regulated in MM and MALAT1 contributed to cell viability, invasion, and glycolysis while inhibited apoptosis through the miR-1271-5p/SOX13 axis in MM cells, which might hold the promise of the treatment for MM patients at the molecular level.

The oncogenic role of MALAT1 in the progression of human cancers has gradually emerged. For instance, Zhang et al. announced that MALAT1 accelerated the progression of renal cell carcinoma via the regulation of the miR-203/BIRC5 axis (Zhang et al. 2019). Sun et al. claimed that MALAT1 expression was elevated and regulated cell proliferation and apoptosis in ovarian cancer through directly targeting miR-503-5p (Sun et al. 2019). Si et al. asserted that MALAT1 could activate autophagy and refrain apoptosis in colorectal cancer via sponging miR-101 (Si et al. 2019). Besides, MALAT1 was shown to be overexpressed in MM patients (Cho et al. 2014). Liu et al. alleged that down-regulation of MALAT1 restrained cell proliferation and motivated apoptosis of MM cells (Liu et al. 2017). Consistently, we also discovered the up-regulation of MALAT1 in MM serum samples and cells. After MALAT1 was knocked down, cell viability, and invasion were repressed but apoptosis was enhanced, implicating the oncogenic role of MALAT1 in MM.

Proverbially, glycolysis is a representative oxygen-independent biochemical metabolic pathway, in which glucose is converted into pyruvate to accumulate lactate, accompanying with the generation of ATP (Akram 2013; Ganapathy-Kanniappan 2018). Hence, glucose consumption, lactate production and the ratio of ATP/ADP are usually deemed as the indexes of glycolysis (Liu et al. 2016). In addition, a number of enzymes are involved in the glycolysis process, including HK2, GLUT1, LDHA, and so on (Wan et al. 2017; Xu et al. 2017). During the current report, MALAT1 knockdown decreased the glucose consumption, lactate production, and ratio of ATP/ADP, as well as the down-regulation of HK2 and GLUT1 levels, suggesting MALAT1 generated a promoted effect on glycolysis process.

LncRNAs function as miRNA sponges to regulate the progression of various cancers generally (Cui et al. 2017; Liu et al. 2018). Here, miR-1271-5p was identified as a miRNA target of MALAT1. MALAT1 could directly inhibit the expression of miR-1271-5p in MM cells. Furthermore, MALAT1 knockdown-induced effects on MM cells were all returned by miR-1271-5p inhibition, indicating that MALAT1 played its oncogenic function via sponging miR-1271-5p in MM cells. miRNAs usually combine with target mRNAs to modulate various cellular behaviors (Cho 2007; Matoulkova et al. 2012). SOX13 was proved to be a downstream gene of miR-1271-5p. The up-regulation of SOX13 in MM during our study was in accordance with the previous finding (Xu et al. 2018). In addition, SOX13 relieved the miR-1271-5p-induced inhibition of cell viability, invasion, and glycolysis but the promotion of apoptosis in MM cells. MiR-1271-5p acted as a tumor repressor by targeting SOX13 in MM.

Furthermore, MALAT1 could affect SOX13 expression via the negative interaction with miR-1271-5p in MM cells. The regulatory network of lncRNA-miRNA-mRNA is in the elucidation of multiple human cancers (Fan et al. 2018; Song et al. 2018; Tang et al. 2019). For example, MALAT1 regulated cisplatin resistance through the miR-101-3p/VEGF-C axis in bladder cancer (Liu et al. 2019a) and the hindering of MALAT1/miR-199a/ZHX1 axis suppressed the progression of glioblastoma (Liao et al. 2019). Therefore, the role of MALAT1 in MM was achieved by miR-1271-5p/SOX13 axis, which was verified by further experiments in vivo. Inhibition of MALAT1 reduced tumor growth of MM by increasing miR-1271-5p and decreasing SOX13 in vivo, insinuating that MALAT1 contributed to tumorigenesis via the regulatory axis of miR-1271-5p/SOX13.

To conclude, the MALAT1 level was up-regulated in MM patients and cells and MALAT1 promoted tumorigenesis, invasion, and glycolysis through the regulation of miR-1271-5p/SOX13 axis. The MALAT1/miR-1271-5p/SOX13 modulatory network provided a neoteric perspective for the development of MM and MALAT1 might be a critical therapeutic and diagnostic target for MM. These fruitful works might lay a foundation for the molecular therapy of MM.

## Data Availability

The analyzed data sets generated during the present study are available from the corresponding author on reasonable request.
